# Host-driven citrullination of extracellular vesicle–derived microbial proteins links *P* *orphyromonas gingivalis* to rheumatoid arthritis

**DOI:** 10.1080/20002297.2026.2751754

**Published:** 2026-10-20

**Authors:** Xuefei Cheng, Weichen Gong

**Affiliations:** a Department of Microbiology and Immunology, Nihon University School of Dentistry at Matsudo, Chiba, Japan; b Laboratory of Animal Food Function, Graduate School of Agricultural Science, Tohoku University, Sendai, Japan; c Livestock Immunology Unit, International Education and Research Center for Food and Agricultural Im-munology (CFAI), Graduate School of Agricultural Science, Tohoku University, Japan

**Keywords:** *Porphyromonas gingivalis*, rheumatoid arthritis, host-driven citrullination, outer membrane vesicles, citrullinated autoantigen

## Abstract

**Background:**

Rheumatoid arthritis (RA) is closely linked to citrullinated antigens and HLA-DRB1 risk alleles, yet the origin of immunogenic citrullinated peptides remains incompletely understood. While *Porphyromonas gingivalis* has been implicated in RA through its bacterial peptidylarginine deiminase (PPAD), previous studies using PPAD-deficient *P. gingivalis* in mouse RA models reported that PPAD deletion did not attenuate disease development, suggesting the existence of additional RA-promoting mechanisms. Here, we computationally evaluate a complementary mechanism in which host peptidylarginine deiminase 4 (PADI4) may modify *P. gingivalis*-derived proteins, generating microbial neoantigens with preferential presentation by RA-associated HLA alleles.

**Methods:**

We developed a computational framework integrating known PADI4 and PPAD substrate preferences with MHC-II antigen presentation prediction. Human proteins were first stratified based on their susceptibility to PADI4 and/or PPAD-mediated citrullination, and their presentation profiles across RA risk-associated (HLA-DRB1*04:01 and 04:04) and protective (HLA-DRB1*13:01 and 13:02)alleles were analyzed through MHC-II binding prediction.

**Results:**

This analysis revealed distinct presentation signatures, including (a) global risk enrichment, (b) high-affinity binding bias, (c) characteristic allelic presentation ratios. Application of these three features led to the identification of five candidate proteins with potential relevance to RA risk, including gingipain R1 and four outer membrane proteins.

**Conclusions:**

These findings propose a hypothesis that host PADI4-mediated citrullination of *P. gingivalis* proteins may represent an additional mechanism contributing to *P. gingivalis*-associated acceleration of RA pathogenesis.

## Graphical abstract

**Figure:**
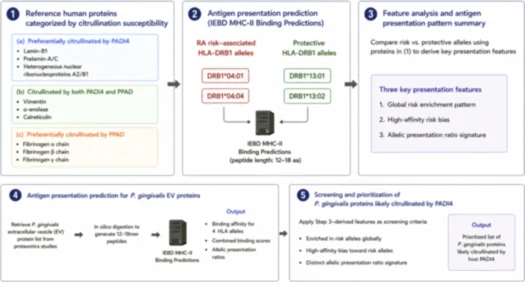

## Introduction

Rheumatoid arthritis (RA) is a chronic autoimmune disease characterized by persistent synovial inflammation and the production of autoantibodies against citrullinated proteins [1]. Protein citrullination, a post-translational modification catalyzed by peptidylarginine deiminases, has been widely implicated in the generation of neoantigens that break immune tolerance [2]. Among these enzymes, PADI4 has been extensively studied and is strongly associated with RA susceptibility, particularly through its role in modifying host proteins such as vimentin, *α*-enolase, and calreticulin [3–6]. Citrullinated vimentin is one of the most well-characterized autoantigens in RA, with anti–mutated citrullinated vimentin antibodies correlating with disease severity and joint damage [7]. Similarly, *α*-enolase has been identified as a major citrullinated autoantigen, and antibodies against citrullinated *α*-enolase peptides (e.g. CEP-1) have been linked to both genetic risk factors and environmental triggers such as smoking [8]. Calreticulin has also been reported to undergo citrullination and contribute to immune activation, potentially through its roles in antigen presentation and modulation of innate immune signaling [9]. These citrullinated proteins are frequently targeted by anti-citrullinated protein antibodies (ACPAs), which are highly specific for RA and are thought to play a central role in disease pathogenesis.

In addition to host-derived citrullination, microbial factors have been proposed to contribute to RA pathogenesis. Among microbial sources of citrullination, Porphyromonas species have attracted particular attention because several members encode bacterial peptidylarginine deiminase (PPAD)-like enzymes. Although PPAD was initially considered unique to *P. gingivalis*, subsequent studies identified conserved and secreted PPAD homologues in other Porphyromonas species, including *P. gulae* and *P. loveana*. Notably, PPAD in these species is predominantly associated with outer membrane vesicles (OMVs), suggesting that vesicular transport may represent a conserved mechanism for delivering bacterial citrullinating activity. Nevertheless, *P*. gingivalis remains the most extensively investigated species in the context of RA, whereas the clinical relevance of PPAD-producing species such as *P*. gulae remains less clear [10,11]. PPAD-mediated modification of host proteins, particularly fibrinogen, has been proposed as one potential mechanism linking periodontitis to RA [12]. Biochemical studies have shown that *P. gingivalis* can process host proteins through gingipain activity and subsequently generate citrullinated peptides via PPAD, including epitopes derived from fibrinogen, vimentin, *α*-enolase, and calreticulin [12–14]. These findings suggest that PPAD may contribute to the generation of immunogenic citrullinated neo-epitopes capable of triggering ACPA responses. However, evidence from experimental models suggests that PPAD alone may not fully account for the pro-arthritogenic effects of *P. gingivalis*. In a mouse model of experimental arthritis, oral inoculation with *P. gingivalis* exacerbated joint inflammation and increased citrullinated protein levels, whereas the use of a PPAD-deficient mutant did not result in a clear attenuation of disease severity or systemic inflammatory responses. Notably, host-derived PAD enzymes, including PADI4, remained detectable in joint tissues and were not significantly altered by PPAD deletion [15]. Given that PADI4 is highly expressed in inflammatory environments and capable of modifying a broad range of substrates [16], we hypothesized that *P. gingivalis*-derived proteins—particularly those released through OMV—may serve as substrates for host PADI4. Such a mechanism would provide an alternative route for the generation of citrullinated microbial neoantigens, thereby contributing to RA pathogenesis through a PPAD-independent pathway. *P. gingivalis* should not be regarded simply as an infectious agent whose presence is synonymous with periodontal infection. Rather, it is currently considered a periodontal pathobiont and keystone species that may be detected in both periodontally healthy individuals and patients with different stages of periodontal disease. Periodontitis is now understood as a chronic inflammatory disease arising from dysbiotic microbial communities and a susceptible host response, rather than from infection by a single bacterial species. Accordingly, throughout this study, the potential contribution of *P. gingivalis* to RA is considered in the context of dysbiotic colonization and exposure to its virulence factors and extracellular products [17,18].

These observations raise a critical unresolved question: what are the relevant antigenic substrates that sustain citrullination-driven autoimmunity in RA? While several host proteins have been identified as targets of PADI4, recent proteome-wide studies have revealed that PADI4-mediated citrullination is far more widespread than previously appreciated [19]. Notably, large-scale citrullinome analyses have identified thousands of citrullination sites across diverse classes of proteins, yet only a limited subset has been convincingly linked to RA pathogenesis [19]. This discrepancy suggests that the mere presence of citrullination is insufficient to explain disease-specific autoimmunity. Instead, additional layers of selection—particularly antigen processing and presentation—are likely required to determine immunological relevance.

Consistent with this notion, genetic studies have identified the major histocompatibility complex (MHC) class II region as the strongest risk locus for RA, with specific HLA-DRB1 alleles playing a central role in shaping disease susceptibility [20]. Variants encoding the so-called ‘shared epitope,’ particularly HLA-DRB1*04:01 and 04:04 [21,22], are strongly associated with increased risk of ACPA-positive RA, whereas alleles such as HLA-DRB1*13:01 and 13:02 confer protective effects [23–25]. These alleles differ in their peptide-binding grooves and are thought to influence both the repertoire and efficiency of antigen presentation, thereby shaping downstream adaptive immune responses. Together, these findings suggest that antigen presentation, rather than citrullination alone, may represent a critical bottleneck in determining which antigens become immunologically relevant in RA.

In principle, the pathogenic potential of a given protein would be best evaluated by integrating both its susceptibility to citrullination and its capacity to be presented by disease-associated MHC-II molecules. However, predicting the precise sites of citrullination remains challenging due to the context-dependent nature of this modification, which often requires experimental validation. Therefore, alternative strategies that bypass explicit site prediction while capturing immunological relevance are needed. In parallel, microbial proteins represent a largely unexplored source of potential antigens, particularly in the context of systemic dissemination. Bacterial OMVs have emerged as important mediators of host–microbe interactions, capable of transporting diverse bacterial proteins beyond the site of periodontal niche [26]. OMVs released by *P. gingivalis* can enter the circulation and deliver a repertoire of microbial proteins to distal tissues, thereby providing a plausible route for microbial antigens to access host enzymatic machinery and immune surveillance systems. Importantly, *P. gingivalis* OMVs are not passive carriers of randomly packaged bacterial proteins. Proteomic and functional studies have demonstrated enrichment of major virulence-associated components, including gingipains, hemagglutinins, and fimbrial proteins, and have further shown that fimbrial expression can influence both OMV production and host-cell invasion. Thus, OMVs provide a biologically relevant vehicle through which multiple *P. gingivalis* antigens and virulence factors may be simultaneously exposed to host tissues and immune surveillance. This feature is particularly relevant to RA because several OMV-associated proteins, especially gingipains, have independently been associated with RA-related immune responses [27].

To address these challenges, we developed a computational framework that leverages antigen presentation as a filtering layer to prioritize candidate antigens. Rather than relying on predefined citrullination sites, our approach is based on the primary amino acid sequences of proteins and their intrinsic capacity to be presented by MHC-II molecules. By comparing peptide binding preferences between RA-risk and protective HLA-DRB1 alleles, we aimed to identify candidate microbial antigens that are preferentially presented in a disease-associated immunogenetic context.

Previous clinical, biochemical, and experimental studies have already established substantial evidence linking *P. gingivalis*, gingipains, PPAD-mediated citrullination, and RA-associated autoimmunity. Therefore, the objective of the present study was not to propose *P. gingivalis* as a previously unrecognized contributor to RA. Rather, we sought to address a distinct unresolved question: whether *P. gingivalis* OMV proteins can be systematically prioritized according to their compatibility with host PADI4-mediated modification and RA-associated HLA antigen presentation. We therefore developed an integrative computational framework to identify microbial proteins displaying convergent RA-risk HLA presentation signatures and to evaluate whether such candidates overlap with proteins previously implicated by experimental or clinical evidence (Figure 1).

**Figure 1.**
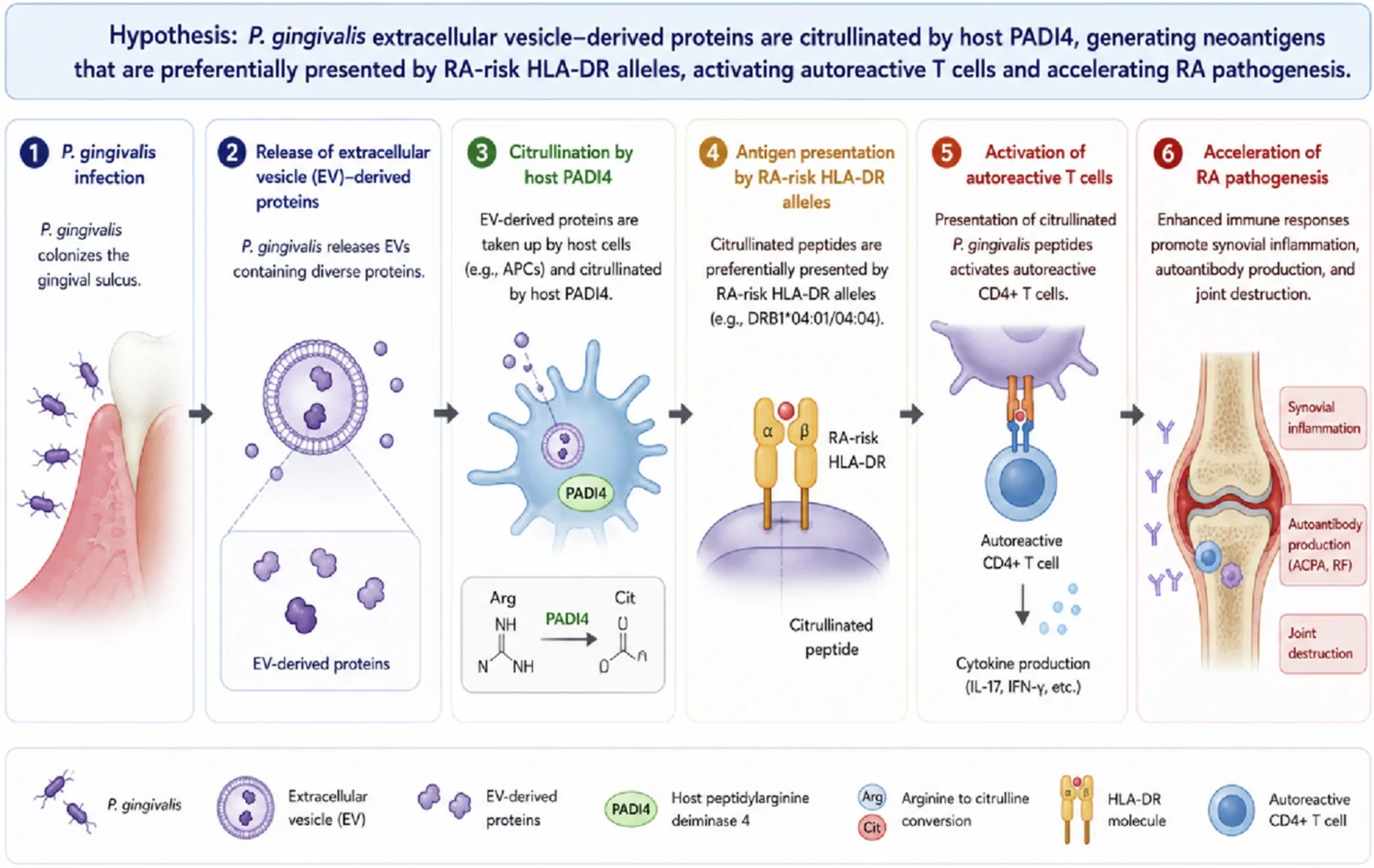
Proposed model linking host PADI4-mediated citrullination of *P. gingivalis* proteins to RA pathogenesis. Schematic illustration of the proposed mechanism by which *P. gingivalis* contributes to RA through host-mediated citrullination of microbial proteins.

## Results

### Comparative analysis of antigen presentation patterns between PADI4- and PPAD-mediated citrullination

Building on the observations above, we next sought to systematically examine how different sources of citrullination may influence antigen presentation in RA. To this end, we stratified proteins into three categories based on their predominant citrullination source: (i) proteins primarily modified by host PADI4 (encoded by LMNB1, LMNA, HNRNPA2B1) [19], (ii) proteins subject to both PADI4- and PPAD-mediated citrullination (encoded by VIM, ENO1, CALR), and (iii) proteins predominantly citrullinated by bacterial PPAD (fibrinogen *α*, *β*, and *γ* chains).

Notably, proteins within the PADI4-dominant group, including LMNB1, LMNA, and HNRNPA2B1, are functionally enriched in essential nuclear and regulatory processes. LMNB1 and LMNA encode lamin B1 and lamin A/C, respectively, which are key structural components of the nuclear lamina and play critical roles in maintaining nuclear architecture, chromatin organization, and genome stability [28]. These proteins are also involved in regulating DNA replication, transcription, and mechanotransduction, highlighting their fundamental role in cellular homeostasis [29]. In parallel, HNRNPA2B1 encodes a heterogeneous nuclear ribonucleoprotein that is central to RNA metabolism, including pre-mRNA splicing, mRNA transport, and stress response regulation [30]. Importantly, large-scale proteomic analyses have identified these proteins as major substrates of PADI4-mediated citrullination, particularly in the context of transcriptional regulation and RNA processing [19]. Given their involvement in core cellular functions, citrullination of these proteins is likely to represent a physiologically regulated process rather than a disease-specific event. This may explain why peptides derived from the PADI4-dominant group do not exhibit a clear bias toward presentation by RA-associated HLA-DRB1 risk alleles. In contrast to proteins associated with inflammation or extracellular exposure, these nuclear and RNA-binding proteins may be less prone to generating immunodominant epitopes, thereby limiting their contribution to pathogenic ACPA responses. Consistent with this notion, we observed that peptides derived from the PADI4-dominant group did not exhibit a clear or preferential binding pattern toward RA-risk HLA-DRB1 alleles, suggesting that endogenous citrullination alone may not be sufficient to drive disease-specific antigen presentation.

Given that proteins predominantly modified by PADI4 alone, including LMNB1, LMNA, and HNRNPA2B1, did not exhibit such preferential presentation patterns, suggesting that PADI4-mediated citrullination in isolation is insufficient to drive RA-associated antigenicity. Based on these observations, we next focused on systematically comparing proteins co-modified by PADI4 and PPAD with those predominantly modified by PPAD alone. In this context, PPAD-driven citrullination was used as a reference baseline to evaluate how the additional contribution of host PADI4 reshapes antigen presentation profiles. Then, we found that peptides derived from proteins co-modified by both PADI4 and PPAD displayed a markedly enhanced binding affinity toward the RA-risk allele HLA-DRB1*04:01 (Figure 2A). Furthermore, quantitative comparison of peptide presentation revealed that these dual-modified proteins generated a significantly higher number of peptides presented by RA-risk alleles relative to protective alleles (Figure 2B).

**Figure 2.**
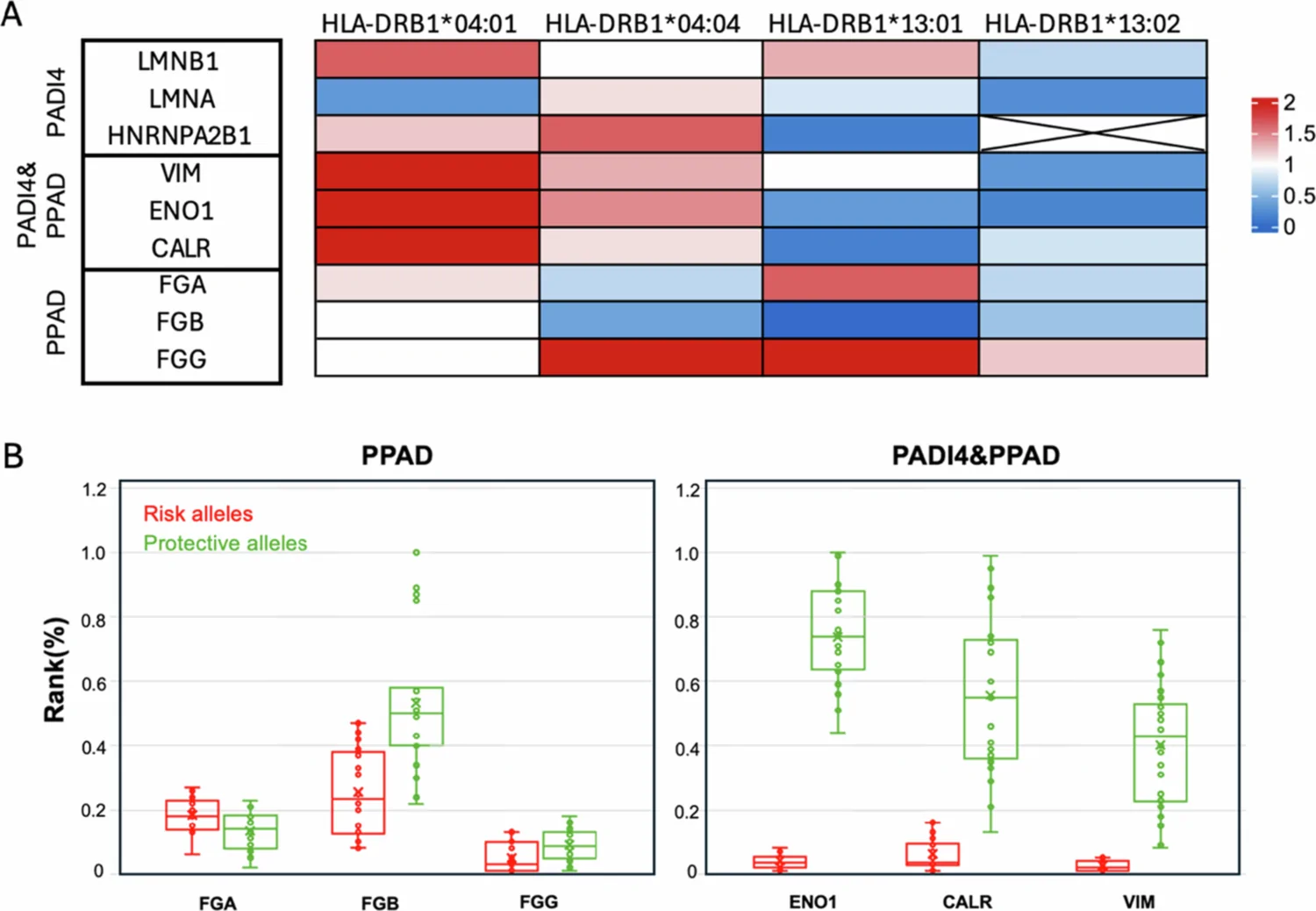
Differential antigen presentation patterns across citrullination-defined protein groups. (A) Heatmap showing predicted antigen presentation patterns of representative proteins categorized by citrullination source: PADI4-dominant (LMNB1, LMNA, HNRNPA2B1), co-modified by PADI4 and PPAD (VIM, ENO1, CALR), and PPAD-dominant (FGA, FGB, FGG). Antigen presentation was evaluated across RA-risk HLA-DRB1 risk alleles (DRB1*04:01, DRB1*04:04) and protective alleles (DRB1*13:01, DRB1*13:02). Color intensity indicates relative binding affinity (IEDB rank-based scoring). PADI4-dominant proteins do not exhibit a consistent risk allele–biased presentation pattern, whereas co-modified proteins show preferential presentation by risk alleles. (B) Distribution of predicted binding affinities for peptides derived from PPAD-dominant proteins (left) and PADI4&PPAD co-modified proteins (right). Risk-associated alleles (red) and protective alleles (green) are compared. Co-modified proteins display a clear enrichment of high-affinity peptides presented by risk alleles, while PPAD-dominant proteins do not show a comparable bias.

Collectively, these results suggest that the interplay between host- and microbe-mediated citrullination may critically influence antigen presentation landscapes. Rather than acting independently, PADI4 may modulate PPAD-derived substrates to generate a distinct pool of neo-epitopes with enhanced presentation by RA-associated HLA alleles, thereby providing a potential mechanistic basis for the amplification of autoimmune responses.

### Development of a systematic framework to identify risk-enriched antigen presentation signatures

To systematically identify antigens preferentially presented by RA-associated alleles, we established a multi-criteria screening framework based on three complementary dimensions: (a) global risk enrichment pattern, (b) high-affinity risk bias, and (c) allelic presentation ratio signature.

To enable quantitative and reproducible prioritization, explicit thresholds were defined for each dimension. For the global risk enrichment pattern, peptides predicted to bind MHC-II molecules with high confidence (IEDB rank ≤ 1%) were considered, and candidate antigens were required to exhibit a ≥80% proportion of presentations by RA-risk alleles (Figure 3A). For the high-affinity risk bias, the top 25 highest-affinity peptides were required to be exclusively presented by risk alleles (Figure 3B). For the allelic presentation ratio signature, each RA-risk allele was compared individually with each protective allele. Significant risk-biased presentation was observed in three comparisons within the PADI4&PPAD co-modified protein group, whereas the PPAD-dominant group did not show comparable differences. The mean values from these significant PADI4&PPAD-derived ratios were therefore used as reference thresholds for downstream screening of *P. gingivalis*-derived proteins (Figure 3C).

**Figure 3.**
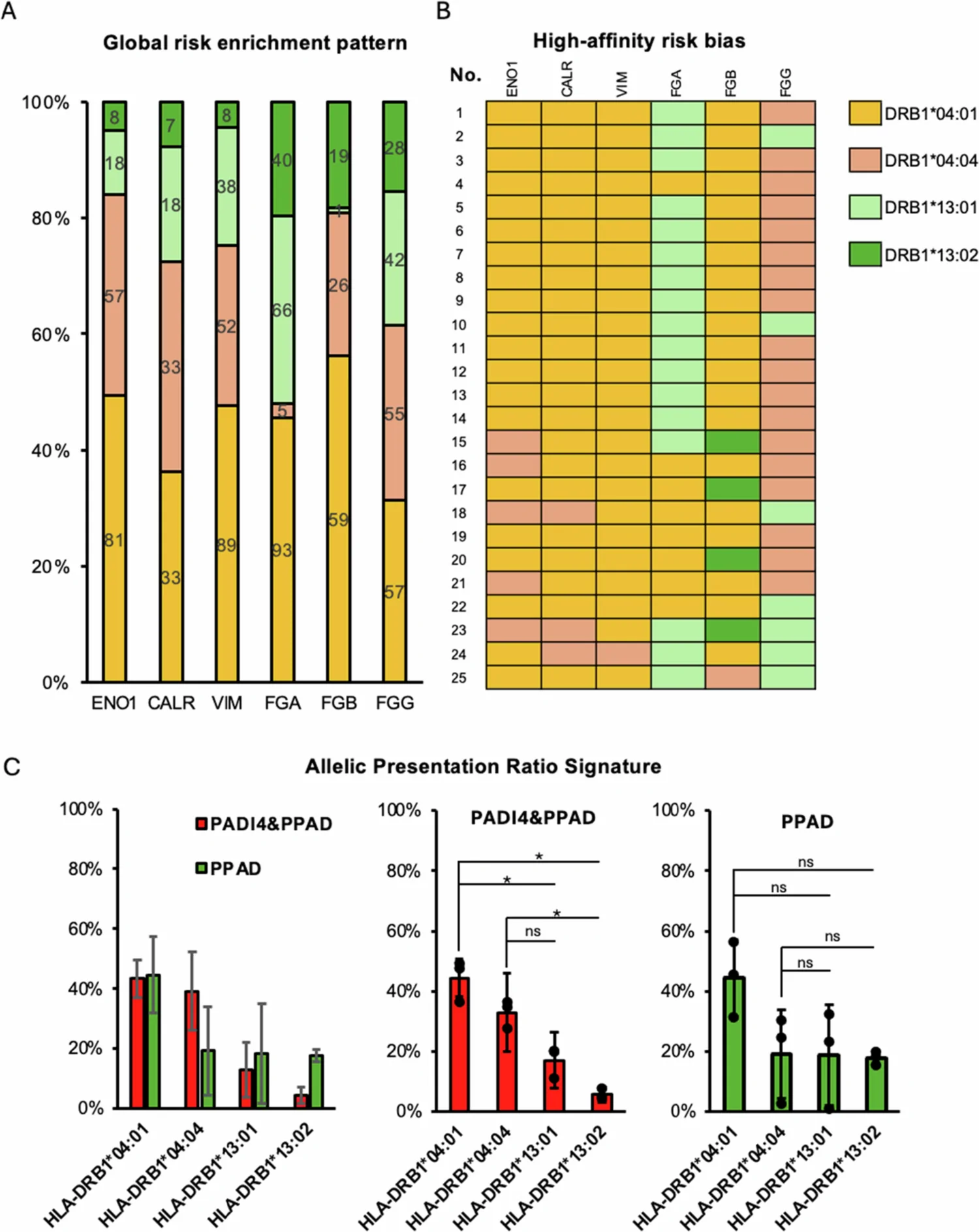
Multi-layered HLA-dependent antigen presentation framework reveals risk-biased signatures and enables candidate selection criteria. (A) Global risk allele enrichment patterns across representative antigens. Stacked bar plots show the proportion of predicted antigen presentation events attributed to individual HLA-DRB1 alleles for each protein. Risk-associated alleles (DRB1*04:01 and DRB1*04:04) dominate the presentation landscape for citrullination-independent proteins (ENO1: *α*-enolase, CALR: calreticulin, and VIM: vimentin), whereas citrullination-dependent proteins (FGA, FGB, and FGG: fibrinogen *α*, *β*, and *γ* chains) display a more balanced or protective allele–enriched distribution. These results indicate a global risk allele bias specific to citrullination-independent antigens. (B) High-affinity risk bias in top-ranked antigen presentation. Heatmap representation of the top 25 predicted peptide–HLA binding events for each protein. Each row corresponds to one of the highest-affinity presentation events, and columns represent individual HLA alleles. Citrullination-independent proteins exhibit a strong dominance of risk-associated alleles among top-ranked binders, with minimal contribution from protective alleles. In contrast, citrullination-dependent proteins show more heterogeneous allele representation, indicating reduced specificity toward risk alleles at the high-affinity level. (C) Left panel: Overall distribution of predicted antigen presentation proportions across HLA-DRB1 alleles for proteins co-modified by PADI4 and PPAD (red) and PPAD-dominant proteins (green). Middle panel: Quantitative comparison of presentation ratios for PADI4&PPAD co-modified proteins across individual HLA alleles. Risk-associated alleles (HLA-DRB1*04:01 and 04:04) show significantly higher presentation proportions compared to protective alleles (HLA-DRB1*13:01 and 13:02). Right panel: Corresponding analysis for PPAD-dominant proteins, which do not exhibit significant differences between risk and protective alleles. Statistical significance between risk-associated alleles and protective alleles was evaluated using unpaired two-tailed Student's *t*-tests. *P* < 0.05 was considered statistically significant (*, *P* < 0.05; ns, not significant).

These thresholds were derived from the antigen presentation profiles of known RA-associated host proteins and were subsequently applied to prioritize *P. gingivalis*-derived proteins with a high likelihood of undergoing host PADI4-mediated citrullination.

### Identification of *P. gingivalis*-derived proteins with convergent risk-associated presentation features

We next applied this framework to proteins derived from *P. gingivalis* OMVs, given that OMVs can disseminate systemically from periodontal sites and thereby provide a plausible mechanistic link between the dysbiotic periodontal niche and RA pathogenesis. To ensure robustness and reproducibility, we analyzed previously reported OMV proteomic datasets rather than performing *de novo* protein identification [31].

Across the *P. gingivalis* OMV proteome, substantial heterogeneity in predicted antigen presentation profiles was observed. Application of the global risk enrichment criterion (≥80% risk allele presentation among peptides with IEDB rank ≤ 1%) identified 18 candidate proteins (Figure 4). Among these, 16 proteins further satisfied the high-affinity risk bias criterion, with the top 25 predicted binders exclusively associated with RA-risk alleles (Figure 5). Subsequent evaluation using the allelic presentation ratio signature refined the candidate set to 8 proteins that exhibited consistent risk-biased presentation patterns relative to protective alleles (Figure 6). Integration across all three criteria ultimately yielded five proteins (UniProt IDs: B2RM93, F5X8K0, B2RHG4, OMP41, and Q7MTV9) that simultaneously satisfied all selection thresholds (Figure 7A,B).

**Figure 4.**
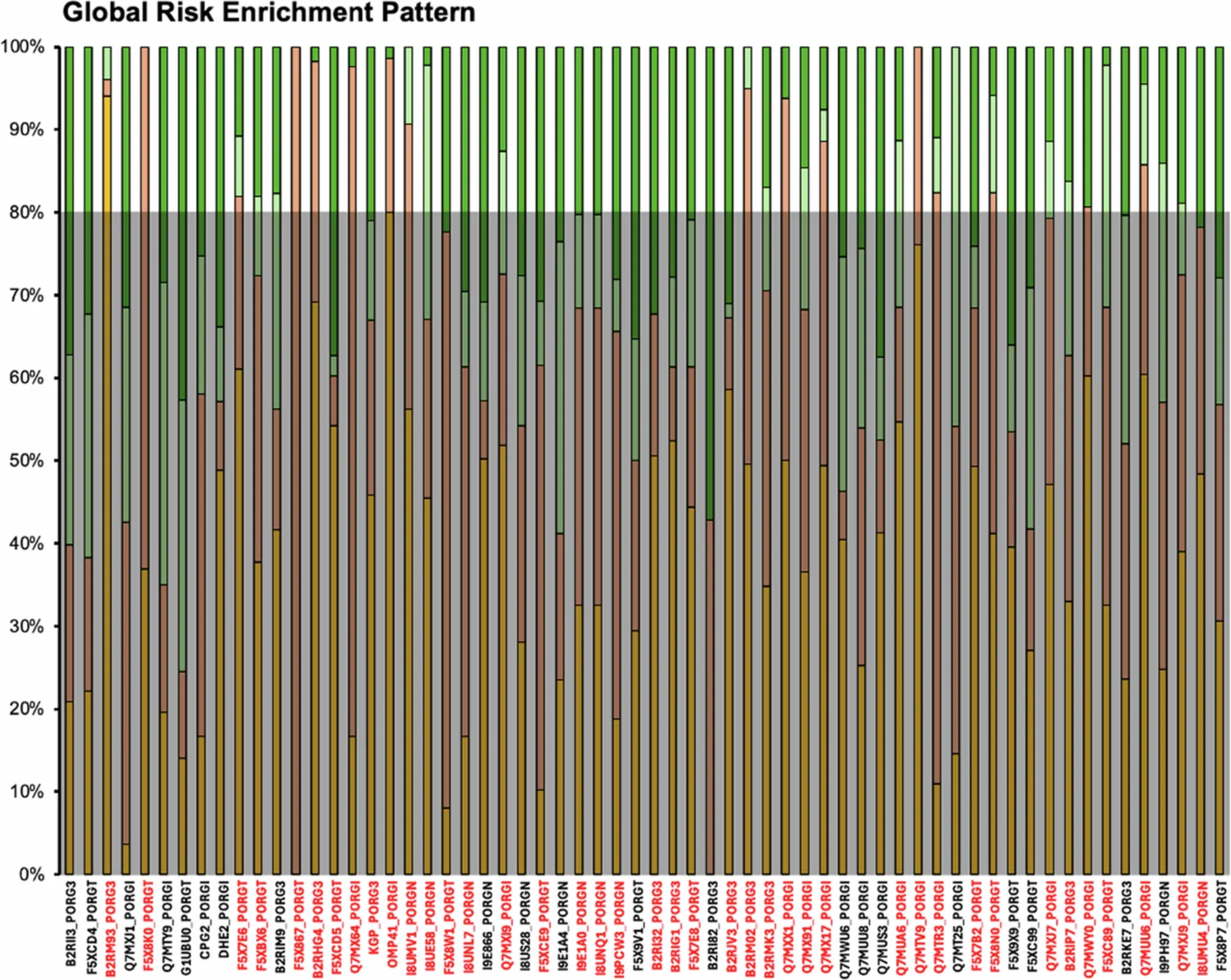
Global risk allele enrichment patterns across proteins derived from *Porphyromonas gingivalis* OMVs. Stacked bar plots show the proportion of predicted antigen presentation events attributed to individual HLA-DRB1 alleles for each OMV protein. A substantial subset of proteins exhibits enrichment of presentation events in risk-associated alleles (DRB1*04:01 and 04:04), exceeding the predefined threshold (shaded region), indicating a global risk-biased presentation pattern.

**Figure 5.**
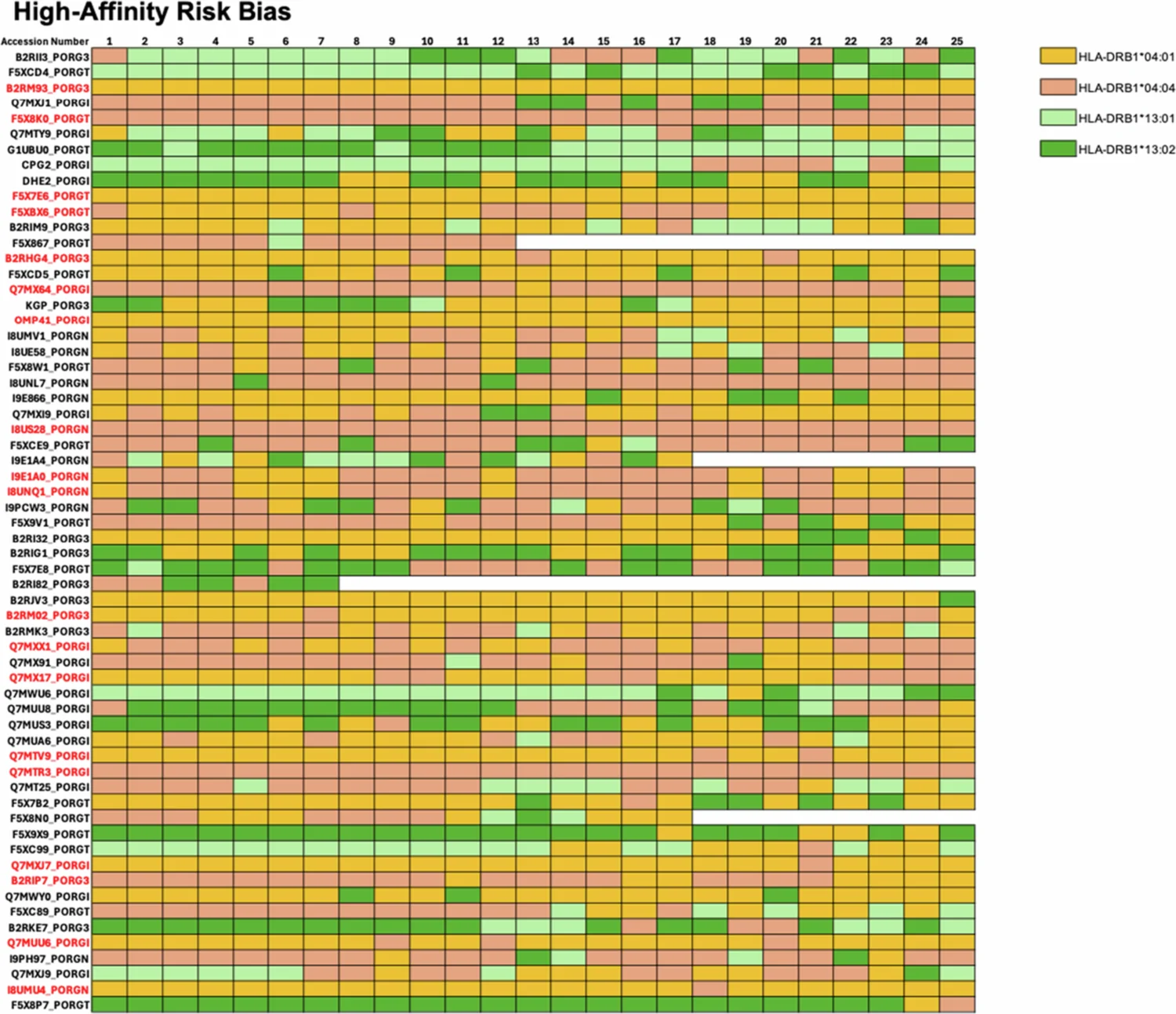
High-affinity risk bias among top-ranked peptide–HLA interactions. Heatmap showing the distribution of the top 25 predicted binding events for each OMV protein across HLA alleles. Each row corresponds to an individual protein, and columns represent ranked high-affinity binding events. Proteins displaying a dominant contribution from risk-associated alleles among top-ranked binders are highlighted (red labels), consistent with a high-affinity risk bias.

**Figure 6.**
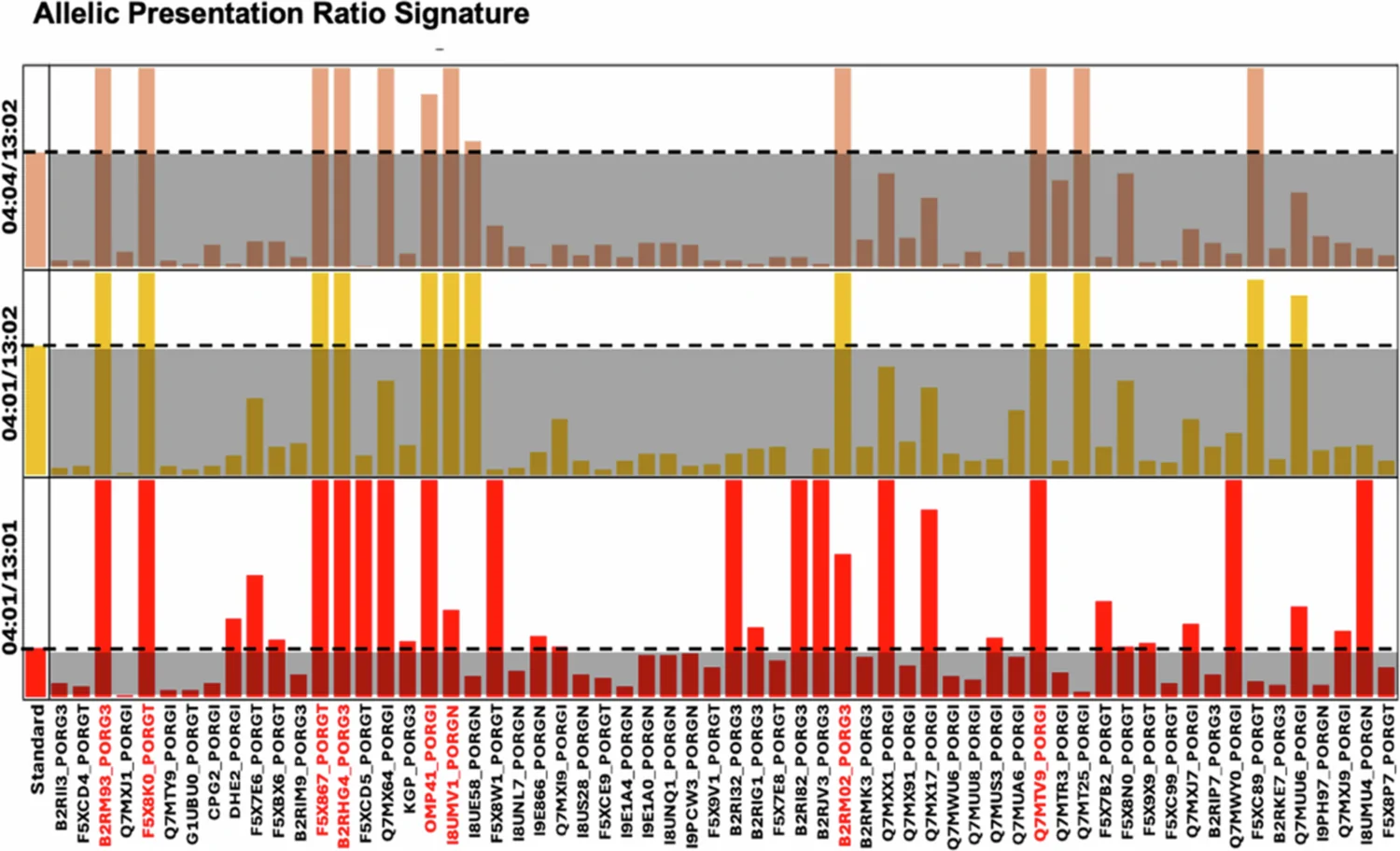
Allelic presentation ratio signatures across OMV proteins. Bar plots illustrate the ratios of antigen presentation between risk-associated and protective alleles for each protein under different allele comparisons. Proteins exceeding the predefined ratio thresholds (dashed lines) are identified as exhibiting a risk-skewed presentation profile. Consistent enrichment of risk allele presentation across multiple ratio comparisons further refines candidate selection.

**Figure 7.**
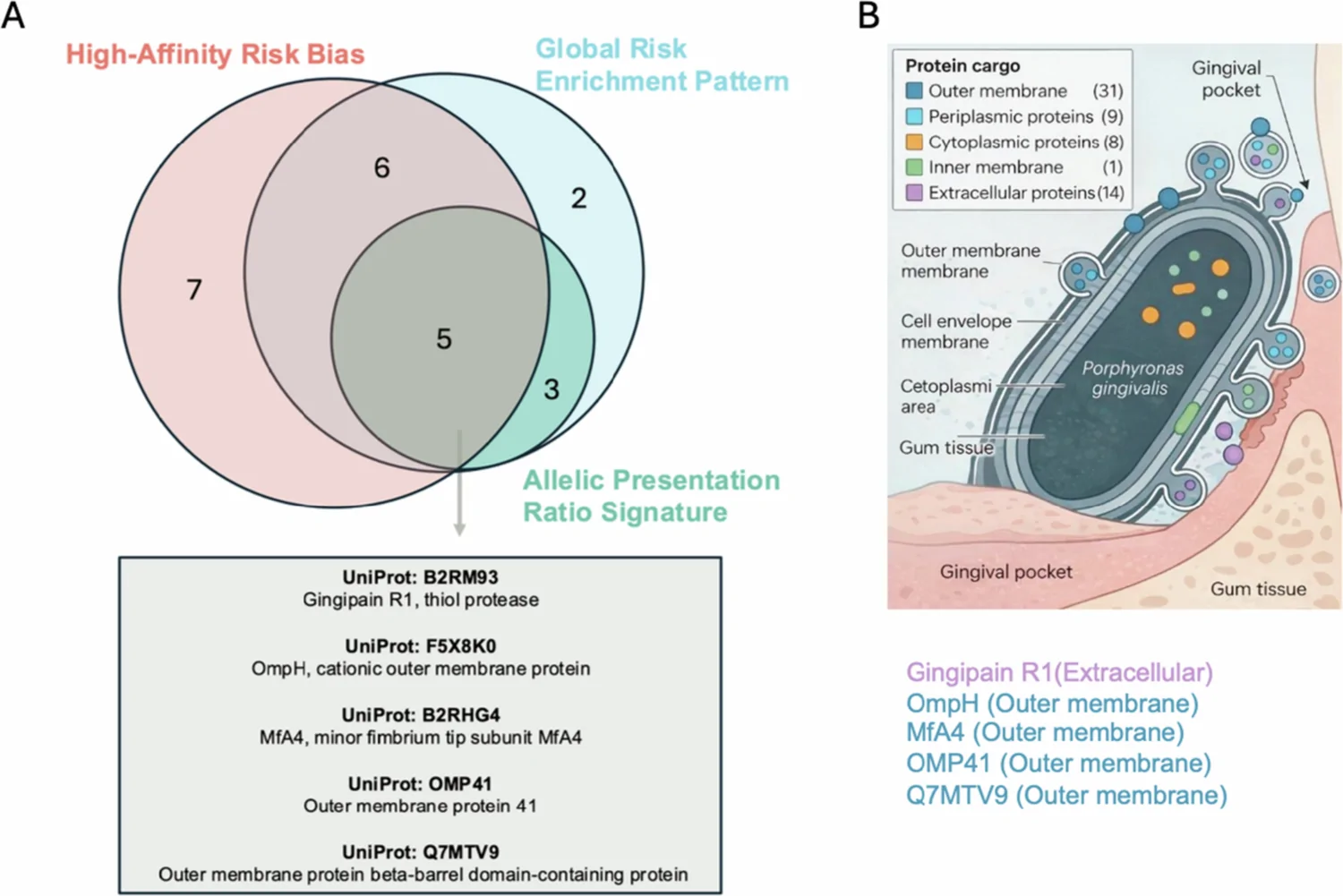
Application of the HLA-dependent antigen presentation framework identifies candidate *P. gingivalis* OMV proteins with risk-biased presentation signatures. (A) Venn diagram illustrating the overlap among proteins satisfying the three screening features: global risk enrichment pattern, high-affinity risk bias, and allelic presentation ratio signature. A total of five proteins were consistently identified at the intersection of all three criteria. The corresponding UniProt IDs and protein annotations are listed below, including gingipain R1 (B2RM93), OmpH (F5X8K0), Mfa4 (B2RHG4), OMP41, and Q7MTV9. (B) Schematic representation of *P. gingivalis* and its OMV release, highlighting the localization of the five prioritized proteins. Four proteins are annotated as outer membrane components, while gingipain R1 is an extracellularly secreted protease. The distribution of these proteins across bacterial compartments supports their accessibility to the host immune system following OMV-mediated dissemination.

Notably, four of the five prioritized proteins were annotated as outer membrane-associated proteins (F5X8K0, B2RHG4, OMP41, and Q7MTV9), whereas the remaining candidate was gingipain R1 (RgpA; B2RM93), an arginine-specific cysteine protease and major virulence factor of *P. gingivalis*. The identification of RgpA is particularly notable because gingipains are abundant and biologically active components associated with *P. gingivalis* OMVs. Beyond their general roles in bacterial virulence, gingipains may be mechanistically relevant to citrullination by cleaving host proteins at arginine residues and thereby generating C-terminal arginine-containing substrates accessible to PPAD. In addition, humoral responses against gingipains have been repeatedly associated with RA. Increased antibodies against arginine gingipains have been reported in individuals before the clinical onset of RA, while combined anti-RgpA and anti-PPAD antibody responses have also been investigated as serological indicators of RA. Thus, the independent prioritization of RgpA by our HLA-based computational screening is consistent with existing clinical and biochemical evidence linking gingipain-associated antigen exposure to RA [32,33].

Together, these findings highlight a convergent subset of OMV-associated proteins that are not only systemically accessible but also exhibit strong bias toward presentation by RA-risk HLA-DRB1 alleles, supporting their potential role as substrates for host PADI4-mediated citrullination and contributors to RA-relevant immune responses.

### Risk allele–biased antigen presentation is associated with peptide-level and physicochemical features

To further investigate the underlying basis of risk allele–biased presentation, we analyzed peptide-level features of the identified OMV-derived proteins. Among the five candidate proteins identified, we selected three representative proteins (B2RHG4, OMP41, and Q7MTV9) exhibiting the highest number of peptides presented by risk alleles and the strongest binding affinities for subsequent analysis of antigen presentation patterns.Heatmap analysis revealed that peptide presentation is not uniformly distributed across HLA alleles but instead exhibits a strongly skewed repertoire, with distinct subsets of core peptides selectively enriched in RA-associated alleles. This selective enrichment is not merely quantitative but reflects a qualitative filtering process, whereby risk alleles preferentially capture a specific subset of peptides that are largely absent or weakly presented by protective alleles (Figure 8A). Consistent with this observation, binding affinity distributions revealed that risk-associated alleles are enriched for high-affinity binders, forming a distinct binding landscape characterized by a concentration of top-ranked peptides. In contrast, protective alleles display a broader and more diffuse affinity distribution, suggesting reduced selectivity and weaker peptide stabilization (Figure 8B).

**Figure 8.**
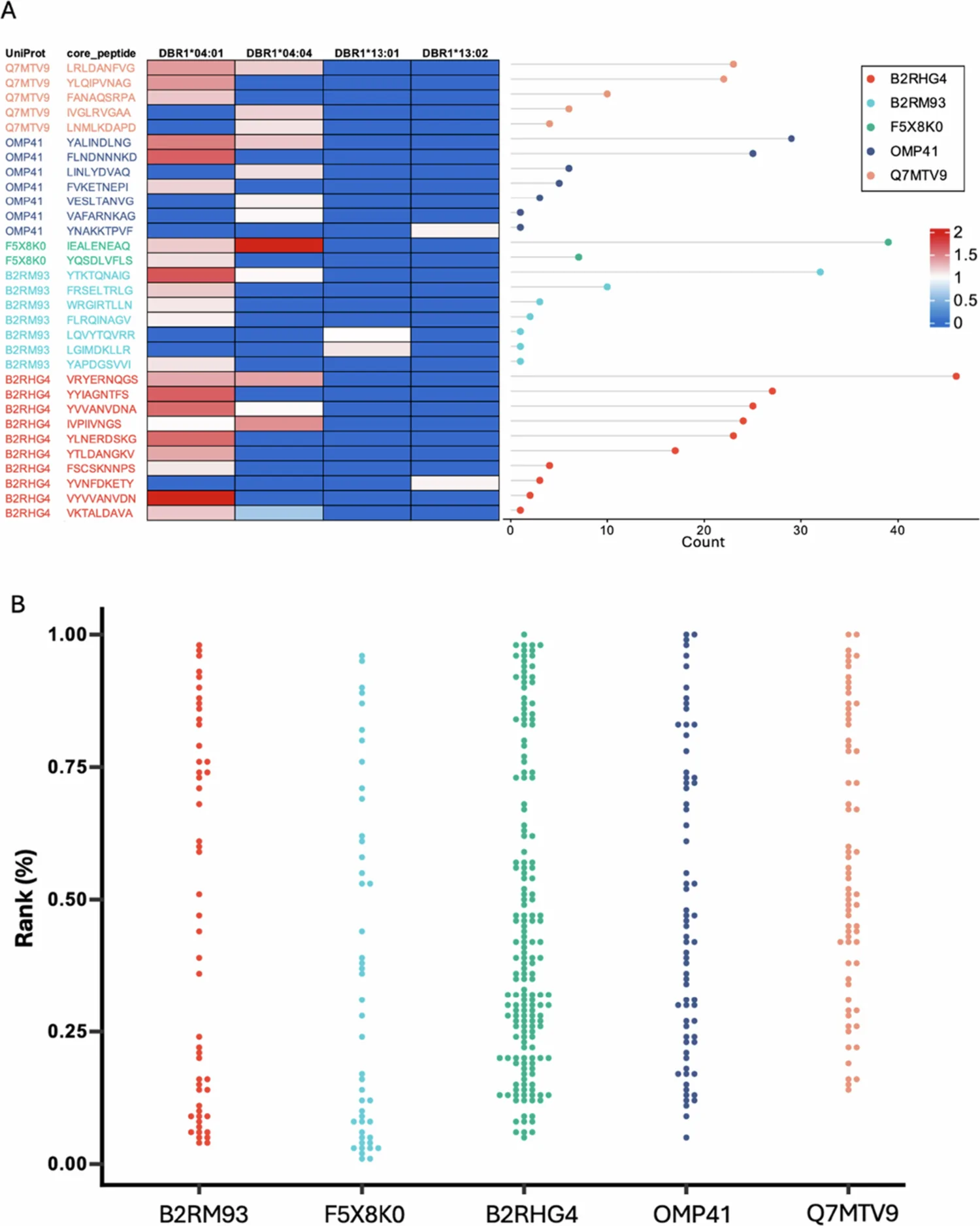
Risk allele–biased antigen presentation is associated with distinct peptide composition in OMV-derived proteins. (A) Allele-specific antigen presentation profiles of core peptides derived from candidate OMV proteins. Heatmap showing the predicted binding affinity (rank score) of representative core peptides across HLA-DRB1 alleles. Risk-associated alleles (DRB1*04:01 and 04:04) exhibit preferential presentation of specific peptide subsets. The accompanying dot plot summarizes the frequency (count) of peptide presentation events restricted to risk-associated alleles, highlighting differences in presentation breadth among OMV-derived antigens. (B) Distribution of binding affinities for OMV-derived peptides under risk-associated alleles. Scatter plot showing the rank (%) distribution of predicted peptide–HLA binding for each candidate protein under risk allele conditions.

To explore whether these differences are associated with intrinsic peptide properties, we performed amino acid composition analysis of presented core peptides. Amino acid composition analysis demonstrated that peptides preferentially presented by risk alleles share a conserved physicochemical profile, characterized by enrichment in hydrophobic and polar residues. Notably, this compositional pattern was not restricted to bacterial peptides but closely mirrored that observed in host-derived antigens (Figure 9A). Strikingly, comparative analysis between bacterial OMV-derived peptides and host-derived peptides revealed a high degree of physicochemical convergence across multiple residue classes. This convergence suggests that microbial peptides may inherently satisfy the structural and binding requirements favored by RA-associated HLA alleles, effectively mimicking host antigen properties at a physicochemical level rather than through strict sequence homology (Figure 9B,C).

**Figure 9.**
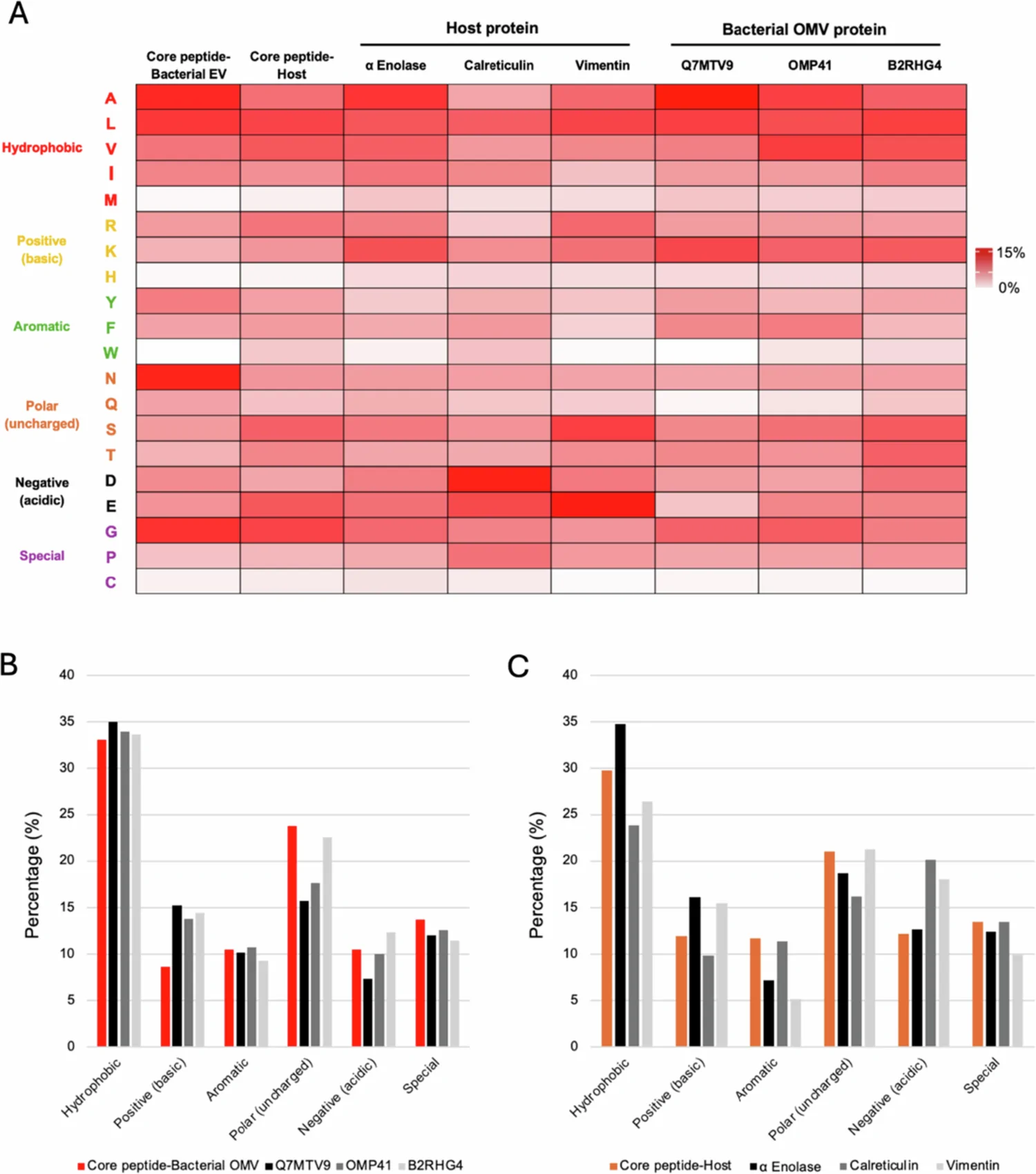
Amino acid composition and physicochemical profiles of core peptides derived from bacterial OMV proteins and host proteins. (A) Amino acid composition profiles of core peptides derived from bacterial OMV proteins and host proteins. Heatmap illustrating the percentage composition of amino acid categories (hydrophobic, positively charged, aromatic, polar uncharged, negatively charged, and special residues) in core peptides. (B) Amino acid composition of core peptides derived from bacterial OMV proteins. (C) Amino acid composition of core peptides derived from host proteins.Bar plots showing the amino acid distribution of host-derived peptides.

These results suggest that risk-associated HLA-DRB1 alleles do not merely recognize specific peptide sequences but instead impose a selective pressure favoring peptides with defined physicochemical characteristics. Importantly, the observed convergence between bacterial OMV-derived peptides and host-derived peptides indicates that microbial antigens may intrinsically mimic host-like binding properties.

Such physicochemical mimicry provides a potential physicochemical basis for cross-recognition, whereby peptides derived from *P. gingivalis* proteins—particularly following host PADI4-mediated citrullination—may display HLA-binding characteristics that partially resemble those of selected self-antigens. This convergence may facilitate the breakdown of immune tolerance and promote aberrant immune activation in RA.

## Discussion

In this study, we developed a systematic computational framework to identify microbial antigens with preferential presentation by RA-associated HLA-DRB1 alleles and applied it to *P. gingivalis* OMV proteins. The association between *P. gingivalis*, protein citrullination, and RA has been extensively investigated, and our study does not seek to redefine this established biological relationship. Instead, the novelty of the present work lies in introducing antigen presentation as an additional computational selection layer that integrates microbial protein exposure with RA-associated HLA genetics. By integrating multiple complementary criteria—including global risk enrichment, high-affinity binding bias, and allelic presentation ratio signatures—we prioritized a small subset of candidate proteins exhibiting convergent risk allele–biased presentation patterns. Notably, the identification of gingipain R1 among the final candidate proteins provides additional support for the biological relevance of our computational framework. Gingipains have been extensively implicated in RA-associated mechanisms, including the processing of host proteins such as fibrinogen and *α*-enolase and the generation of immunogenic neo-epitopes [33]. The fact that our unbiased screening strategy independently prioritized gingipain R1 suggests that the framework is capable of capturing functionally relevant antigens rather than arbitrary candidates. While this observation does not constitute direct validation, it provides an important layer of external consistency, indicating that the identified selection criteria may reflect biologically meaningful features associated with RA-related antigen presentation. This concordance between computational prediction and prior experimental observations strengthens confidence in the utility of the framework for prioritizing candidate microbial antigens.

The prioritization of RgpA is particularly informative in light of the substantial experimental and clinical literature linking gingipains to RA. Gingipains are among the major virulence factors associated with *P. gingivalis* and its OMVs and have traditionally been considered functionally coupled with PPAD-mediated citrullination. By cleaving proteins at arginine residues, arginine-specific gingipains can generate substrates that subsequently undergo PPAD-mediated citrullination, thereby facilitating the production of potentially immunogenic modified peptides. Importantly, the relevance of gingipains extends beyond this biochemical interaction. Humoral responses against *P. gingivalis* gingipains have been detected in RA cohorts and, notably, elevated anti-RgpB antibodies have been observed years before the onset of clinical RA symptoms. Moreover, combined antibody responses against RgpA and PPAD have been proposed as serological indicators associated with RA. These observations provide an important clinical context for our computational finding: RgpA was prioritized not because gingipains were predefined as RA-related proteins, but because its predicted peptide repertoire independently satisfied the HLA-dependent selection criteria established in our analysis. This convergence between computational prioritization and previous biochemical and patient-based observations supports the biological plausibility of the screening framework [12,27,33–35].

A key finding of this study is that risk-associated HLA-DRB1 alleles do not merely recognize specific peptide sequences but appear to preferentially present peptides sharing defined physicochemical characteristics. The observed convergence between bacterial OMV-derived peptides and host-derived peptides suggests that antigenicity in RA may be driven by physicochemical compatibility with HLA binding preferences rather than strict sequence identity. This provides a plausible basis for molecular mimicry, whereby microbial peptides can be presented in a host-like manner and potentially contribute to the breakdown of immune tolerance.

Building on these observations, we propose a conceptual model in which host PADI4-mediated citrullination acts as a critical modifier of microbial antigenicity. *n* this model, *P. gingivalis* releases OMVs containing diverse protein cargo that can disseminate beyond the periodontal niche and interact with host immune cells. Upon uptake by antigen-presenting cells, these microbial proteins become substrates for host PADI4, leading to the conversion of arginine residues into citrulline and the generation of modified peptides with altered binding properties.

Importantly, our results suggest that these citrullinated peptides are not randomly presented but instead preferentially fit the binding landscape of RA-associated HLA-DRB1 risk alleles. This selective presentation may amplify the visibility of microbial-derived neoantigens within the adaptive immune system. As a consequence, peptides derived from *P. gingivalis* proteins may be recognized in a manner analogous to self-antigens, promoting activation of autoreactive CD4⁺ T cells and driving downstream inflammatory responses.Taken together, this model provides a mechanistic framework linking microbial exposure, host enzymatic modification, and HLA-restricted antigen presentation. Rather than acting solely through pathogen-derived enzymes such as PPAD, *P. gingivalis* may contribute to RA pathogenesis through a host-driven pathway in which PADI4 reshapes microbial antigenicity to favor autoimmune recognition. In this model, *P. gingivalis*-derived proteins—particularly those disseminated via OMVs—may serve as substrates for host PADI4, leading to the generation of citrullinated peptides that preferentially fit the binding landscape of RA-associated HLA alleles. In contrast to the traditional PPAD-centric view, this framework highlights a complementary pathway in which host-driven modification of microbial proteins contributes to disease-relevant antigen presentation. Such a mechanism may help explain why PPAD deficiency alone does not fully abolish the pro-arthritogenic effects of *P. gingivalis* [19].

This study has several limitations. First, our analysis is based on computational prediction of antigen presentation and does not directly demonstrate in vivo processing, citrullination, or T cell recognition of the identified peptides. Second, the precise citrullination sites on microbial proteins remain unresolved, and our framework infers PADI4 susceptibility based on presentation patterns rather than direct biochemical evidence. Third, our analysis focuses specifically on the PADI4 pathway and does not account for the potential contributions of other peptidylarginine deiminases or alternative post-translational modification systems that may also influence antigenicity.

Despite these limitations, our work provides a generalizable framework for systematically linking host-driven post-translational modification to microbial antigen presentation. This approach can be extended to other microbial species, including bacteria and viruses, as well as to other immune-mediated diseases beyond RA. More broadly, our findings suggest that the interaction between host modification enzymes and microbial proteins may represent an underappreciated axis in shaping disease-associated antigen landscapes.

Future studies combining experimental validation, site-specific citrullination mapping, and immunological assays will be essential to further test this model. In particular, integrating mass spectrometry–based citrullinome profiling with HLA peptidomics and T cell activation assays may help determine whether the predicted peptides indeed function as immunogenic epitopes *in vivo*.

## Conclusion

In conclusion, this study establishes an integrative computational framework for prioritizing *P. gingivalis* OMV-derived proteins according to their predicted compatibility with RA-associated HLA antigen presentation. Application of three complementary selection criteria—global risk allele enrichment, high-affinity risk bias, and allelic presentation ratio signatures—identified five candidate microbial proteins, including RgpA and four outer membrane-associated proteins. The independent identification of RgpA, a virulence factor already supported by biochemical and patient-based evidence in RA, provides external biological consistency for the screening strategy. Rather than proposing a new association between *P. gingivalis* and RA, our findings refine the existing model by suggesting that host-driven modification and HLA-dependent selection may determine which microbial proteins are most likely to acquire immunological relevance. These candidates now provide a focused set of targets for experimental validation by site-specific citrullination analysis, HLA peptidomics, ACPA recognition, and T-cell activation assays.

## Methods

### Study design

This study was designed as a computational, hypothesis-driven analysis to investigate whether host PADI4-mediated citrullination of *P. gingivalis*–derived proteins could contribute to RA-associated antigen presentation. The overall workflow consisted of three major steps. First, reference human proteins with known susceptibility to PADI4- and/or PPAD-mediated citrullination were curated and categorized to establish a baseline for antigen presentation patterns. Second, MHC-II antigen presentation profiles were predicted for these reference proteins across RA-associated and protective HLA-DRB1 alleles, and key presentation features were systematically derived. Third, these features were applied as screening criteria to *P. gingivalis* OMV proteomic datasets to prioritize candidate microbial proteins with risk allele–biased presentation patterns and potential susceptibility to host PADI4-mediated modification.

All analyses were performed *in silico* using publicly available datasets and prediction tools, and no experimental validation was conducted in this study.

### MHC-II antigen presentation prediction

Epitope presentation was assessed using the IEDB Analysis Resource (https://tools.iedb.org/mhcii/, accessed April 10th, 2026). Prediction Method: NetMHCIIpan 4.1 EL(recommended epitope predictor-2023.09). Peptide length: 12-18 mer sliding window. Analysis: MHC-II binding prediction. Classification: Peptides with Rank ≤ 1% were designated strong binders. Alleles evaluated included RA risk-associated and RA-protective MHC-II variants, based on genetic epidemiology literature [36].

### Comparative visualization of peptide–HLA binding distributions and peptide–HLA interaction maps

To provide a systems-level view of peptide–HLA binding patterns, predicted MHC-II binding ranks were summarized and visualized in two complementary formats. First, for risk-associated HLA alleles, all predicted peptide binders derived from the candidate microbial proteins were grouped by source protein and visualized according to binding rank (%) distribution. Lower rank values indicate stronger predicted binding. These data were plotted as point-based distribution charts to compare the range and density of predicted binders across proteins and alleles.

Second, peptide–HLA interaction maps were generated separately for risk-associated and protective HLA alleles. In this analysis, only peptide–allele pairs with predicted binding rank ≤ 1 were retained in order to focus on the strongest predicted interactions. When multiple binding predictions were present for the same peptide–allele pair, the minimum rank value was retained as the representative score. For visualization, rank values were transformed as −log10 (rank), such that higher values indicate stronger predicted binding. The resulting peptide–HLA matrices were rendered as heatmaps.

All visual summaries were prepared using Microsoft Excel (Version 16.107). Distribution plots were generated from Excel-based data summaries and further arranged graphically in Microsoft PowerPoint (Version 16.107). Heatmaps were generated directly in Excel using the transformed −log10(rank) values.

### Computational and statistical analysis

All analyses were descriptive and computational, performed using Python-based pipelines. Results represent *in silico* predictions and should be interpreted as hypothesis-generating. For the comparisons shown in Figure 3 C, unpaired two-tailed Student's *t*-tests were performed to compare the predicted presentation proportions between individual HLA-DRB1 alleles. Specifically, comparisons were conducted between HLA-DRB1*04:01 and HLA-DRB1*13:01, HLA-DRB1*04:01 and HLA-DRB1*13:02, HLA-DRB1*04:04 and HLA-DRB1*13:01, and HLA-DRB1*04:04 and HLA*-*DRB1*13:02. A *P-*value < 0.05 was considered statistically significant. All other analyses were descriptive and intended for hypothesis generation.

## Data Availability

All data generated or analyzed during this study are included in this published article. The *Porphyromonas gingivalis* outer membrane vesicle (OMV) proteomic dataset used in this study was obtained from previously published publicly available data [31]. Additional data are available from the corresponding author upon reasonable request.
